# Farther Account of a Case of Mollities Ossium
*See Vol. VI. page 288.


**Published:** 1787

**Authors:** W. Goodwin

**Affiliations:** Surgeon at Earl Soham, in Suffolk


### VII. *Farther Account of a Cafe of Mollities Offium* *. *By Mr.* W. Goodwin, *Surgeon at Earl Soham, in Suffolk. Communicated in a Letter to Dr.* Hamilton, *Phyfician at Ipfwich, and by him to Dr.* Simmons.

THE extraordinary foftnefs of the bones in the cafe of Mary Bradcock, of Dalinghoe, near Wickham market, in Suffolk, concerning which I did myfelf the pleafure of writing to you in Auguft, 1785, has been rendered much more fingular fince by a variety of circumftances, with an account of which I now beg leave to trouble you.

At the date of my former account fhe was in the fixth month of her ninth pregnancy, and had been confined to her bed near twelve months. At the ufual period fhe was delivered of a healthy male child that lived fifteen weeks; and being enabled, by the benevolence of the humane perfons who contributed to her relief, to procure all the comforts her forlorn ftate admitted, fhe regained a better ftate of health than fhe had known for fome time before.

During the fpring of 1786 fhe continued in good health and fpirits; but complained at

* See Vol. VI. page 288.

 times times of pain flying from bone to bone. About the beginning of April ſhe again became pregnant, but had no alarming ſymptoms till Auguſt, when the pain of her bones increaſed rapidly, and thoſe which had been broken in 1785 began to ſeparate where they had united with as great, or even more, pain than at their firſt breaking. This excruciating pain, which ſhe ſuffered for ſeveral days previous to the diſſolution of the callus, rendered her continually feveriſh from the irritation, and ſhe declined haſtily in health and appetite.

Violent pain now ſeized freſh parts of the bony ſyſtem, which, after a continuance of ſix or ſeven days, was ſufficient to occaſion new fractures, viz. of three ribs, and of each arm above and below the elbow, making, together, ſeven fractures, which, with the eight that happened in 1785, and the diſſolution of their union the year following, make no leſs than twenty-three fractures which this unhappy woman ſuffered within the ſpace of about two years and a half, and all without any violence, and chiefly while confined to her bed, in which ſhe paſſed the whole of the laſt year of her life, laying conſtantly on her left ſide. You will be pleaſed to obſerve alſo, that in 1785

the the pain continued feveral weeks before a fracture took place, but that of late a few days were fufficient to difpofe the bones to give way.

She died on the 19th of December laft, aged four and thirty years. Her bones, when examined after death, were found to be fo extremely foft, that even thofe of her arms could be eafily cut through with a fmall penknife. The bones of the cranium had not efcaped the effects of the difeafe, as they could eafily be indented with the preffure of a finger. Of all the bones, thofe of the lower extremities had fuffered the leaft, and but little foftnefs was obfervable in them: the back bone, on the contrary, was a good deal affected, for it was nearly as foft as cartilage.

With fome difficulty the byeftanders were perfuaded to permit me to take off the left arm at the fhoulder. This I fhall keep for a few days for the infpection of the curious in the country, and fhall then fend it * to your friend, Dr. Simmons, to elucidate and prove the general truth of my narrative.

* It is now in the poffeffion of Mr. Hunter, to whom we are indebted for fome valuable obfervations on this curious difeafe of the bones, which the reader will find in the next article. — EDITOR.

It

It was obſerved in the former account*, that ſeveral of the patient's family had been afflicted with ſcrophula, but ſhe herſelf had no ſymptoms of that diſeaſe externally. How far her extraordinary ſufferings might be owing to any acrimony of that kind affecting the bony ſyſtem, I will not pretend to determine.

*Earl Soham,*

January 3d, 1787.

